# Remarkable Cure by Phosphorus

**Published:** 1884-08

**Authors:** 

**Affiliations:** Alvensleben, Germany


### CLINICAL BUREAU.

---

### REMARKABLE CURE BY PHOSPHORUS.

DR. BUCHMANN, ALVENSLEBEN, GERMANY.

[*Translated from "Allgemeine Homœop. Zeitung," Vol.* 108, *No.* 13–16. *By B. Fincke, M. D., Brooklyn, N. Y.*, 1884.]

"This pure experience shows throughout that the dose of the homœopathically selected remedy can never be prepared so small that it should not be still stronger than the natural disease, and potent enough to at least in part overcome, extinguish, and cure it, as long as it is able, immediately after its administration, to cause some though slight preponderance of its symptoms over the disease similar to it."—*Hahnemann Organon*, 5th ed., § 279.

The less the greatness of Hahnemann has been understood, the more willing they have been to pass an abject judgment upon him. Likewise the significance of Hahnemann's experience, laid down in the above-quoted section, and its bearing upon posology has, so far, not been estimated sufficiently. The following instructive case will, however, furnish a new proof of how Hahnemann once more has been in the right.

CASE.—F. B., fifty-four years, a mother who has nursed and lost three sons of from eighteen to thirty years of age, one after another, with tuberculosis, suffered since that time from obstinate constipation, several times causing a *typhlitis stercoralis.* After every death she complained for a considerable time of loss of appetite and sleep; she lost flesh, and her hair turned gray and was falling out. For the last two years after the last death the molar teeth on the left side became loose; the gums in this part were found loose on touch; they bled easily, were inflamed, and suppurated now and then. Since the loosening of the teeth she could not chew on the left side.

From that time, also, amblyopia developed. The accommodation of the eyes was so far interfered with that for reading common type she needed convex glasses of fourteen inches focus, and for distinct vision at further distances glasses of twenty-two inches focus. In spite of her hypermetropia, she could not at far distances see as distinctly as formerly. On sewing and reading, pains in the eyes, heat of the face, and pain in the vertex. The stitches and letters ran together, so that she had to give up these occupations. During a four-weekly use of the sea bath at Misdroy she gained a good appetite. The bowels moved once every day and the accommodation of the eyes had very much improved. This salutary effect of the sea-bathing lasted till two weeks after getting home, when the former condition returned.

In the meantime, in August, 1883, the unexpected engagement of her youngest daughter took place, and the fear in October to be separated from her last child by a great distance had a very depressing effect upon the state of her mind. After the separation at times, even with her glasses, she saw the objects as through a black veil and had to bring them nearer to the eyes in order to see them. Sometimes it was as if a dark shade came before her eyes, so that for several seconds she could not see anything and it became so dark before her eyes that she had to grope around to find out where she was. The fear of getting hopelessly blind caused her to talk of her eye trouble to me and to ask for remedies.

Shortly before I had read, in the article of Dr. Hughes "On the action of Medicaments upon the Eye," in regard to Phosphorus: "It is our leading remedy in simple amblyopia, if produced by exhausting causes, such as excesses *in venere* or tobacco, night watching, grief, etc."

I then consulted the *Materia Medica Pura* in order to compare the totality of the present and former symptoms of the patient with the pathopœsis of Phosphorus.

Those pathogenetic symptoms were perfectly covered by the following Phosphorus symptoms:

Taking cold easily in open air, causing toothache;

Changes of weather he feels by the pains in advance;

Tiredness of the whole body, especially in the thighs;

Great anger at the least trifle;

Frequent falling of the hair;

Pain in the eyes on reading by daylight and in the evening by candle-light;

Vanishing of sight on reading;

She must bring the things near to the eyes if she wants to see them distinctly; in the distance she sees everything like in a smoke or through a black veil, but also in keeping things near she cannot stand it long to see distinctly. She can see better when the pupils are dilated by shading the eyes with her hand;

Like a black veil before the right eye;

Suddenly getting blind, as if a gray cover were before the eyes, several times;

Lessened appetite, with weakness;

Full, hard, tense abdomen, distended by flatus;

Stool with strong pressure and scanty defæcation;

No stool the first days; obstinate constipation for six days;

Hard stool in small knots, covered with slime and some adhering blood;

Stool hard, every two days;

The teeth get so loose that she cannot chew;

The gums bleed readily and are detached from the teeth at the least motion.

Taught by experience that in chronic diseases of the eye high potencies show themselves more efficacious than low ones, I gave to the patient, 1883, November 27th, at 9 A. M.:

Phosphorus Cm (Fincke), three pellets on the tongue, and, besides, allowed the vial to act by induction for ten minutes in her right hand, remembering the extraordinary effect of Lachesis 5M (Fincke) (see HOMŒOPATHIC PHYSICIAN, Vol. III, p. 260).

After three minutes' induction:

Yawning, collection of water in the mouth, and contraction of the jaws, hindering the opening of the mouth; then drawing pains from the lower angles of both shoulder-blades down as far as the renal region, where the pain is worst and lasts longest; frequent eructations.

After five minutes' induction:

Rumbling in the bowels, with urging to stool.

After ten minutes' induction:

Stool easily moved; the rumbling in the bowels continues for fifteen minutes and the urging to stool for one hour; the sensation of weakness in the legs is relieved and she can see without the sensation of a black veil before the eyes.

11.45 A. M., the vial is held in her hand for ten minutes.

After three minutes induction:

Collection of water in the mouth; yawning; sensation of contraction of the jaws hindering the opening of the mouth; good appetite at noon; after eating, continued urging to stool.

2 P. M.—Sensation of weakness in the legs; repeated loud rumbling in the bowels; she feels motion in the bowels: n different abdominal regions; sensation of stiffness in the neck; frequent pressing and urging to stool; till the evening four times fruitless efforts to evacuate; in the night waking up several times with rumbling in the bowels.

Nov. 28th, 1.30 P. M.—Evacuation of hard fæces.

Nov. 29th, 7 A. M.—Evacuation of hard fæces.

Nov. 30.—In the morning after awaking, rumbling in the bowels till 8 A. M., then a pappy evacuation; she can to-day read a type one millimeter high with glasses No. 22, while formerly she has used No. 14 for type three millimeters high. During her sojourn in Misdroy, she had observed the same change. The former tiredness in the morning after rising is gone, likewise the seeing as through a dark veil; appetite improved.

Dec. 1st.—In the morning, in short intervals, two pappy stools. At noon a snow squall after several clear days. At noon again urging to stool with wound-like pain at the anus, rendering walking difficult in the afternoon.

Dec. 2d.—In the morning at 6 A. M., pappy evacuation with rumbling; the aversion to meat is gone; she took at noon with the greatest relish roast goose, and in the evening calf's liver.

Dec. 3d, 6 A. M.—Slight evacuation; took cold yesterday afternoon in driving out, which caused toothache in the evening; disturbed sleep in the night; this forenoon obscuration of sight and momentary blindness.

℞ Phosphorus 45m (Fincke), three pellets on the tongue. Rainy weather.

In the evening shortly before 7 P. M., pappy stool.

Dec. 4th, 6 A. M.—Solid stool; the eye symptoms of yesterday are gone.

Dec. 5th, 6.30 A. M.—Solid stool.

7 P. M.—Pappy stool.

Feb. 10th, 1884.—Till to-day, every morning about 7 A. M. a solid and toward 7 P. M. a pappy evacuation; the eye symptoms did not reappear; she can read several hours without complaint by lamplight; the molar teeth on the left side have grown so firm that she can chew with them again.

EPICRISIS.—On looking upon the facts in the singularly rapid cure of the above-described chronic disease, caused by grief and sorrow, we notice, first, the appearance of yawning, collection ot water in the mouth, and spasm of the jaw after holding the vial for three minutes in the right hand, and later also the same symptoms after holding the vial in the left hand, similar to my observation upon the same person by the inductive process in her proving of Lachesis 5M (Fincke). The symptom mentioned last coincides perfectly with the pathopœtic symptom of Phosphorus 228—" closure of the jaws so that she could not get the teeth asunder"—so that we cannot doubt the appearance of a pathopœtic symptom of Phosphorus in this uncommon complaint, which also returned in the same manner after holding the vial in the left hand for three minutes. The affinity of the current of Phosphorus-ether to the ganglia which supply the salivary glands has set in motion by reflex action the neighboring motor nerves connected by sympathetic fibres as promptly as the pressure upon the key moves the clock-work by inducing the electric current. Also the irritation of sensible nerves is indicated by the peculiar pains in the back, which, indeed, is a symptom common to most remedies in physiological provings.

After five minutes already ensued, the therapeutic action of the remedy in a direction which I had neither expected nor intentioned, the torpid peristaltic action awoke, after having continued for years, and a quite unusual slight evacuation ensued quicker than after any purgative which patient had formerly at times resorted to without any favorable result.

After induction by the left hand pathopœtic effects occurred in the afternoon such as we observe after strong cathartics, and they returned after four days in consequence of a sudden change of weather in a somewhat different manner, which I have observed not infrequently in former physiological provings, and which is characteristic of Phosphorus action. In spite of these intercurrent pathopœtic symptoms of Phosphorus, probably caused by the repeated induction, there appeared daily an evacuation modified by it till after the cessation of these symptoms, since December 5th an alternately harder and softer evacuation without any concomitant complaint occurred each morning and evening toward seven o'clock—an after action, lasting now about three months, an observation perhaps never made before.

How, then, may this quite unusual radical cure of a chronic disease which not seldom resists all medicinal efforts be explained physiologically, a cure which commenced as early as five minutes after the induction of the high potency? It seems very easy to me, by the assistance of my scientific explanation of the homœopathic Law of Simility. Just as alcohol, by greater physical affinity to water, expels the sugar of milk out of its watery solution, and occupies its place; just as Arsenicum, in virtue of its pathopœtic affinity to Phosphorus, surpassing the nutritive affinity, drives the Phosphorus out of the living vitellin in the brain and steps in its place, so *in our case the high potency of Phosphorus by its stronger pathopœtic affinity has* SIMILIA SIMILIBUS *driven out the morbific cause originating from grief, etc., from a certain territory of brain-cells and nerves, and has taken its place.* The acceptability of this explanation must also be evident to our opponents. (Compare *Organon*, §§ 29, 148.)

The apparently favorable success from the action of the sea-salt in Misdroy was a pathopœtic symptomatic effect transiently favoring the organic change (Stoffwechsel), similar to an allopathic purgative by which the morbific cause was not removed but its influence only suspended. (*Organon*, § 38.)

The following may serve for explaining the rapid action upon the previously torpid peristaltic action:

1. Certain diseases of the brain cause constipation.

2. Anxiety and fright produce a noxe which causes the, by Trousseau so called, nervous diarrhœa.

3. Professor Nothnagel thinks it probable that such disturbances of the peristaltic motion in certain neuropathic conditions are mediated along the course of the pneumogastric nerve, since it is the only nerve which passes from the brain to the alimentary canal.

4. It is known that in consequence of anger the biliary secretion passing into the intestines can be so arrested that jaundice results.

5. Fibres of the pneumogastric nerve pass also to the gall bladder, and connect with the offshoots of the sympathetic plexus of the liver.

Though the physiological bearings of the pneumogastric nerve upon the intestinal canal of man are not sufficiently ascertained (*Deutsche Wochenschrift*, 1884, 3), the cause of the disease described and the rapid cure of a constipation of many years' standing seems to furnish the proof that fibres of the pneumogastric regulate not only the respiration and motion of the heart but also the peristaltic motion, and that in our case by removing the morbific cause also a chronic neurosis of the fibres of the pneumogastric which regulate the peristaltic motion has been cured.

By misunderstanding, Hahnemann's doctrine has been exposed to rejection in the eyes of the prejudiced schoolmen, though Homœopathy is able to solve physiological problems much safer than vivisection, with which now such an abuse is carried on.

The relapse of the amblyopia, December 3d, I attribute to the cold taken the preceding day.

From the transient character of the improvement by the influence of the sea-salt it appears that this amblyopia was caused by a neurosis of the concerning ocular nerves and by the disturbed innervation of vasomotoric and trophic fibres. According to the experiment of Nasse (Beneke's *Outlines of the Pathology of the Organic Change*, 1874, p. 225) there is no doubt that not only the greater percentage of water but especially a simultaneous increase of the amount of the chloride sodium of the blood favors the transfusion of the blood-serum through animal membranes. Thus temporarily, by artificial promotion of the diffusion, the amblyopia could have been suspended symtomatically by the increased organic change without simultaneously restoring the normal innervation, while the amblyopia could only be radically cured by a simile which drove out the morbific cause, replacing it *positively*.

To those who favor the view that the remedies satisfy their affinities mostly in the path of nutrition through the circulation it at first sight appears incomprehensible that a high potency should unfold its pathopœtic and therapeutic action more rapidly and vigorously through the walls of the vial and the skin than through the stomach, but we see, *e. g.*, with regard to Quicksilver that the mucous membranes are less penetrable to large doses than the external skin, and we conceive that in the present case the rapid pathopœtic and therapeutic action of Phosphorus cannot be explained in any other way than by the effusion of a current of Phosphorus-ether going out uninterruptedly for several minutes from more than eight thousand pellets through the body, the atoms of which are so far distant from one another that in their shocks they could not experience any impediment. Here indeed we stand before an enigma, but it is no more inexplicable to us than the action of the mineral magnetism to the physicist and the action of the Biod to the physiologist, which at the present day also can only be comprehended by considering it as an emanation of ether.

In regard to the transportability in the rapidity and penetration through solid bodies and to the immediate physiological effect upon the sympathetic nerve, there is such a resemblance between the modus operandi of Phosphorus by induction, and that of Biod, that we can style our high potency from which we have seen such astonishing results as Phosphorus-od, the carriers of which are the sugar pellets.

Further experience must decide whether for such a brilliant cure an especial disposition is required, of course, under the necessary condition of the natural homœopathicity of the remedy.

In our case an explanation of the cure can only be admitted under the supposition of a pure neurosis where anatomical changes as causes are precluded. There is, on account of the great similarity of the symptoms pathogenetic to the symptoms of Phosphorus, not the least doubt that in this case Phosphorus was the homœopathic remedy, and this is the reason why also Phosphorus has proved a curative for amblyopia from similarly acting causes. But besides, between effects of the morbific cause and the pathopœtic effects of Phosphorus upon the intestines, which are only explicable by neurosis of some fibres of the pneumogastric nerve, there is such a similarity that one is almost forced to ascribe to Phosphorus a part in the action of the morbific cause as a constituent of the nerve-mass.

Considering the weak nutritive affinity of Phosphorus in the vitellin of the nerve-cells, to which I have alluded before, we must assume that, in our case, by grief and sorrow as psychical moments pre-eminently retarding the organic change, a noxe has been created having a pathogenetic affinity of certain districts of nerve-cells, for the Phosphate of Lecithin introduced as nutriment, an affinity greater than the nutritive one, by which this has been wakened and restored again after the removal of the noxe. Other noxes of less consistence we have seen to originate in more transient exaltatory emotions, and even, entering the milk, to act pathogenetically upon the nursling, a fact which also is only to be explained by the affinities. We know now by experience that the nutritive affinity to certain substances in the nutriments, combined chemically or physically, is present in the same complexes of cells as their pathogenetic affinity to the same substances, and that pathogenetic disturbances in the pathological equilibrium and in the mode of arrangement of certain inorganic molecules in these complexes cause similar pathogenetic symptoms as the pathopœtic introduction of these inorganic nutritive substances to those complexes.

Schüssler's hypothesis, which has nothing to do with the law of simility, does not seem to me correct. He does not care for the annihilation of the morbific cause, but for the replacement of molecules of an inorganic nutriment which may possibly have been lost, though their substances are daily eliminated in *palpable* quantities by the urinary organs. Daily palpable oscillations in the quantity of these cell-constituents take place without functional disturbance, hence we can only have to deal with a permanent inability of the cells in appropriating certain inorganic substances in sufficient quantity. Should then indeed inorganic cell-constituents in a billionth attenuation be easier assimilated than those offered in the nutriments, they nevertheless would soon, by the continual organic change, be lost again, and not suffice quantitatively under continued massy elimination. Besides, the concerning substances are incessantly conveyed to the cells by the circulation in the most assimilable form in the common articles of diet, and our case shows that by homœopathic annihilation of the morbific cause the elective activity in the sick cell territory has been restored immediately. Nobody will maintain that the possibly lost Phosphorus molecules have been replaced immediately by some pellets of the hundredth thousandth potency of Phosphorus.

Phosphoric acid alone in its organo-chemical combination in the Lecithin of the nutriments has a nutritive affinity to the photoplasma of the nerve-cells forms its integrant constituent, and is in organic combination eliminated with the glycerine in the organic change.

Not even the higher organism of plants is enabled to immediately convey inorganic nutritive matter to the cells. These are carried first into the leaves by the ascending sap-stream and there combined organically under the influence of light before they can be used for the formation of cells predestined for animal nutrition.

The roots of plants also act, *e. g.*, repulsive to the Nitrogen of Ammoniac and of the Potash of the Sulphate of Potash.

The inorganic nutritive substances act in free and uncombined condition pathopœtically by organo-chemical or organo-physical affinity, or by their diffusive equivalent, especially when potentiated, or else they could not have a place in our materia medica.

It cannot be objected that the potency which Schüssler recommends might act pathopœtically. The motto and the present clinical case show that such an action has been exerted, even by the highest potencies.

Dr. Schüssler has the merit of having unintentionally enriched our homœopathic treasure of remedies by some valuable but not sufficiently proved remedies.

Our pathologists have already partly comprehended that they cannot dye the blood-disks by iron, and that they cannot supply the want of phosphate of lime in the cartilages. The goat of Weiske had to pay the experiment with her life. (Beneke U. S., p. 374.)

Supplement.—The anniversary of the death of the patient's sons, about the end of January, had revived the painful remembrance of the lost ones, and she could not free herself from a recapitulation of all the sorrowful events during that unfortunate time. The consequence was: diminished appetite, disturbed sleep, and amblyopia, with the above-described eye symptoms, since a few days. To this state of things came.

February 13th, 1884, continual distention, hardness and tenderness of the abdomen during the day, worse on pressure, and the hitherto regular soft evacuation at 7 P. M. appeared as late as 8 P. M. in smaller quantity, hard and difficult to void.

9.30 P. M.—Took the vial with Phosphorus 45m (Fincke), in her left hand for ten minutes.

After three minutes of induction: Frequent yawning, with interrupted running of water from the nostrils.

After eleven minutes of induction: Frequently repeated eructations, which render the abdomen soft and painless.

After sixteen minutes of induction: Rumbling in the bowels with urging to stool.

After twenty minutes of induction: Sensation of constriction in the vagina (also at the first induction of Phosphorus this symptom had appeared, but was neglected. It is not in Hahnemann's provings).

February 14th.—From 6 to 7 A. M., three soft evacuations. She had not dined at noon, having no appetite.

2.30 P. M.—Trembling; perspiration, and feeling faint; heat in the face; thirst till 7 P. M., when she eats some white bread; leucorrhœa with wound-like sensation at the introitus vaginæ; in the evening, for the first time since December, no evacuation.

February 15th.—Slight hard evacuation at 7 A. M; lassitude causes her to lie down from 10.30 A. M. to 0.30 P. M; then she takes one spoon of potatoes and one of preserved peas; she don't want meat.

Afternoon.—Obscured sight, like fog before the eyes; it gives pain to look through the glasses; great lassitude, with dislike to speak till 7 P. M.; no stool in the evening.

February 16th.—Restless sleep during the night; no stool in the morning; perspiration at the forehead, with faintness; at noon she takes only a cup of broth for dinner.

Afternoon.—Much affected till 6 P. M.

7 P. M.—She eats a veal cutlet and potatoes with relish.

February 17th.—She can see well again; appetite good; evacuation in the morning and evening at 7 o'clock; corroding leucorrhœa; she feels herself strong again.

February 20th.—Till to-day as February 17th.

February 21st.—The leucorrhœa has ceased; the same evacuation as with Hahnemann's prover symptom 544.

*Ceterum censeo macrodosiam esse delendam.* B. F.

